# An Evolutionary Insight into Zika Virus Strains Isolated in the Latin American Region

**DOI:** 10.3390/v10120698

**Published:** 2018-12-08

**Authors:** Diego Simón, Alvaro Fajardo, Pilar Moreno, Gonzalo Moratorio, Juan Cristina

**Affiliations:** 1 Laboratorio de Virología Molecular, Centro de Investigaciones Nucleares, Facultad de Ciencias, Universidad de la República, Iguá 4225, Montevideo 11400, Uruguay; dsimon@fcien.edu.uy (D.S.); afajardo32@gmail.com (A.F.); pmoreno@cin.edu.uy (P.M.); 2 Laboratorio de Organización y Evolución del Genoma, Unidad de Genómica Evolutiva, Facultad de Ciencias, Universidad de la República, Iguá 4225, Montevideo 11400, Uruguay; 3 Laboratorio de Inmunovirología, Institut Pasteur de Montevideo, Mataojo 2020, Montevideo 11400, Uruguay

**Keywords:** Zika, viral evolution, genetic variability, Bayesian analyses

## Abstract

Zika virus (ZIKV) is an emerging pathogen member of the *Flaviviridae* family. ZIKV has spread rapidly in the Latin American region, causing hundreds of thousands of cases of ZIKV disease, as well as microcephaly in congenital infections. Detailed studies on the pattern of evolution of ZIKV strains have been extremely important to our understanding of viral survival, fitness, and evasion of the host’s immune system. For these reasons, we performed a comprehensive phylogenetic analysis of ZIKV strains recently isolated in the Americas. The results of these studies revealed evidence of diversification of ZIKV strains circulating in the Latin American region into at least five different genetic clusters. This diversification was also reflected in the different trends in dinucleotide bias and codon usage variation. Amino acid substitutions were found in E and prM proteins of the ZIKV strains isolated in this region, revealing the presence of novel genetic variants circulating in Latin America.

## 1. Introduction

Zika virus (ZIKV) is an emerging pathogen member of the *Flaviviridae* family, naturally transmitted between *Aedes* spp. mosquito vectors and human/non-human primates, which serve as amplifying hosts in urban and sylvatic cycles, respectively [1]. The ZIKV genome consists of a single-stranded positive sense RNA molecule of about 10.7 kb with two flanking non-coding regions (5′ and 3′NCR), and a single long open reading frame encoding a polyprotein: 5′-C-prM-E-NS1-NS2A-NS2B-NS3-NS4A-NS4B-NS5-3′. This polyprotein is cleaved into capsid (C), precursor of membrane (prM), envelope (E), and seven non-structural proteins (NS) [2]. ZIKV was isolated for the first time in 1947, from the blood of a sentinel Rhesus monkey stationed in the Zika forest, Uganda [3]. Although ZIKV enzootic activity was reported in diverse countries within Africa and Asia, only a few human cases were reported until 2007, when an epidemic occurred in Micronesia [4]. A large ZIKV outbreak took place in French Polynesia during 2013–2014, and then spread to other Pacific Islands [5]. In early 2015, a ZIKV epidemic outbreak took place in Brazil, estimated at 440,000–1,300,000 cases [6]. By January 2016, locally transmitted ZIKV cases were reported by most countries and territories of the American region to the Pan American Health Organization [7].

ZIKV has spread rapidly across the Americas, causing hundreds of thousands of cases of ZIKV disease in this region, as well as microcephaly associated with congenital infection and other neurological disorders [8]. To date, ZIKV has been classified into two major genetic lineages, namely African and Asian. The African lineage is comprised of two groups: The West African (Nigerian cluster) and East African (MR766 prototype cluster) [9,10]. Previous studies have shown that the Asian genetic lineage is responsible for the Pacific Islands and American outbreaks [11,12].

The two viral envelope proteins, prM/M and E, have been used as major targets for vaccine development against ZIKV infections [13], since they are the main determinants for the high stability of ZIKV, and where epitopes for CD4^+^ and CD8^+^ T-cell adaptive immune responses and neutralizing antibodies are located [13,14]. The most promising vaccines currently in trials are the ones that use a combination of both prM and E proteins [13]. A detailed characterization of prM and E proteins of ZIKV strains circulating in all regions of the world are very important for our understanding of the antigenicity and pathogenesis of ZIKV, as well as for the development of suitable ZIKV vaccines.

For all these reasons, a detailed analysis on the molecular evolution of ZIKV populations is of extreme importance to understand the relation among viruses and hosts, viral survival, fitness, and evasion from the host’s immune system [15,16]. In order to gain insight into these matters, we performed a comprehensive phylogenetic analysis of recently isolated ZIKV strains in the Latin American region. Five different genetic clades were observed revealing a process of diversification among the ZIKV strains circulating in this region. Besides, these genetic clades displayed distinct compositional properties. Amino acid substitutions were found in E and prM proteins of the ZIKV strains isolated, revealing the presence of novel genetic variants circulating in the American region.

## 2. Materials and Methods

### 2.1. Sequences

Complete coding sequences of 61 available and comparable ZIKV strains, isolated from humans in the Latin American region, from December 2014 through to August 2017, as well as comparable 10 ZIKV strains isolated in the south region of the USA were obtained from GenBank (available at http://www.ncbi.nlm.nih.gov). For strains, accession numbers, geographic location, and date of isolation, see Supplementary Material Table S1. Only strains for which the day, month, and year of isolation was known were included. Sequences were aligned using MUSCLE [17].

### 2.2. Recombination Analysis

GARD program was used to detect any possible recombination event [18]. There was no evidence of recombination in the dataset.

### 2.3. Bayesian Coalescent Markov Chain Monte Carlo (MCMC) Analysis

To investigate the evolutionary rate and patterns of ZIKV strains circulating in the Latin American region, we used a Bayesian Markov Chain Monte Carlo (MCMC) approach as implemented in the BEAST package v.1.8.0 [19]. We started by identifying the evolutionary model that best fit our sequence dataset by using the FindModel software (available at http://hiv.lanl.gov/content/sequence/findmodel/findmodel.html). Bayesian Information Criterion (BIC), Akaike Information Criterion (AIC), and the log of the likelihood (LnL), indicated that the Tamura-Nei (TN93) + Γ model was the most suitable model (BIC = 41,048.518; AIC = 39,356.515; and LnL = −19,531.228). Importantly, the strict and the relaxed molecular clock models were implemented to test different models (constant population size, exponential population growth, expansion population growth, logistic population growth, and Bayesian Skyline). Statistical uncertainty in the data was reflected by the 95% highest posterior density (HPD) values. By using the TRACER program v1.6 (available at http://beast.bio.ed.ac.uk/Tracer) we assessed the results. Fourty-million generations were needed to obtain convergence, after a burn-in of 4 million steps, which were enough to acquire a suitable sample for the posterior, assessed by effective sample sizes (ESS) with values over 200. The comparison of models was done by measuring AIC in a Bayesian Monte Carlo (AICM) from the posterior output of each of the models using TRACER v1.6 program. Lower AICM values indicated better model fit. The Bayesian Skyline model was the best model to analyze the data (see Appendix A). Through the Tree Annotator program, maximum clade credibility trees were generated, and then visualized in the FigTree program v1.4.2 (available at http://tree.bio.ed.ac.uk).

### 2.4. Compositional Analyses

For each ZIKV, we determined for the whole polyprotein: Dinucleotide observed/expected ratios (dinucleotide bias), relative synonymous codon usage (RSCU) values for each degenerate codon (all triplets excluding AUG, UGG, and stop codons), and amino acid frequencies; as was described in previous work on the genus *Flavivirus* [20]. These compositional analyses were performed using the R package seqinr [21], with count, rho, and AAstat functions. The relationship between compositional variables and samples was obtained using multivariate statistical analyses; principal component analysis (PCA) is a type of multivariate analysis that allows a dimensionality reduction. Compositional properties of each strain included in this study were obtained, and the distribution of these strains in the plane defined by the first two principal axes of a PCA (PC1 and PC2) examined. Major trends within a dataset were determined using measures of relative inertia and sequences ordered according to their position along the different axes, and correlation between the axes and original variables. For the values obtained from the compositional analyses, see Table S2.

### 2.5. Prediction of Exposed Residues and Structural Regions of E and prM Proteins

To identify exposed residues and coiled regions of E and prM ZIKV proteins, we used the BepiPred approach [22]. BepiPred uses hidden Markov algorithms in combination with propensity scale methods to predict epitopes in protein sequences. From a FASTA file as input, the method outputs a GFF file with prediction scores and classifications, given a threshold. We used the BepiPred online server (available at http://www.cbs.dtu.dk/services/BepiPred) with 0.5 as threshold.

## 3. Results

### 3.1. Bayesian Coalescent Analysis of ZIKV Strains Recently Isolated in the Latin American Region

To address the degree of genetic variability and mode of evolution of the ZIKV strains recently isolated in the Latin American region, a Bayesian MCMC approach was employed [17]. The results shown in Table A1 were the outcome of 40 million steps of the MCMC, using the Tamura-Ney (TN93) + Γ model, a relaxed molecular clock, and the Bayesian Skyline model. The date of the most common ancestor to all ZIKV strains isolated in Latin America was estimated to be in early 2014 (95% HPD October 2013 to December 2014), in agreement with recent results [23,24,25]. A mean rate of evolution of 1.21 × 10^−3^ substitutions per site per year (s/s/y) was found for the ZIKV sequences included in these studies (95% HPD 7.55 × 10^−4^ to 1.66 × 10^−3^). This was also in agreement with recent estimations of 1.15 × 10^−3^ s/s/y [23].

**Figure 1:**
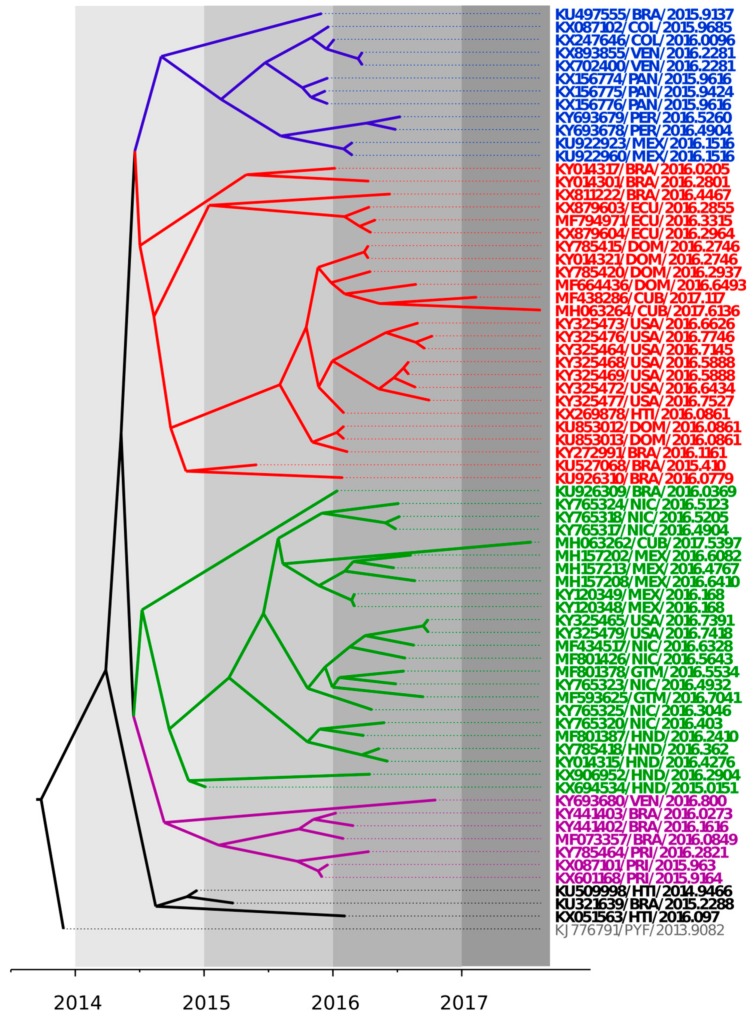
Bayesian maximum clade credibility tree representing the time-scale of ZIKV, obtained by the analysis of 61 complete coding sequences using the Tamura-Ney (TN93) + Γ model, the Bayesian Skyline model, and a relaxed exponential clock. The tree is rooted to the Most Recent Common Ancestor (MRCA) of strains included. The scale at the bottom is in units of evolutionary time and represents the years before the last sampling date. Strains in the tree are shown by their accession number, geographical location, and year of isolation expressed in decimal format. Clades are indicated in blue, red, green, violet, and black.

The phylogenetic relationships among ZIKV strains recently isolated in the Latin American region were explored and summarized in a maximum clade credibility tree shown in Figure 1. ZIKV strains isolated in the Latin American region from 2014 to 2017 clustered in at least five different genetic groups, revealing a significant local genetic diversification of the ZIKV strains isolated in this region. Co-circulation of different genetic lineages was observed in several countries in the region (see for instance the ZIKV strains recently isolated in Cuba in 2017).

### 3.2. Trends in Compositional Properties Across ZIKV Strains Isolated in the Latin American Region

**Figure 2:**
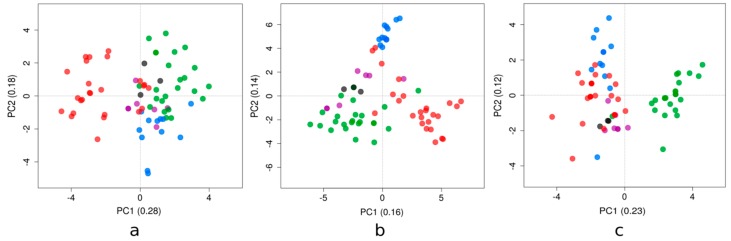
Positions of the ZIKV strains for the first two major axes of principal component analysis of the (**a**) dinucleotide observed/expected ratios, (**b**) relative synonymous codon usage, and (**c**) amino acid frequencies. The proportion of variance explained by each axis is displayed, placed between parentheses. Strains in the plot are colored according to their clade assignment depicted in Figure 1 (i.e., in blue, red, green, violet, and black).

Principal component analysis (PCA) has shown different patterns among the genetic clusters examined (Figure 2; also see Supplementary Material Figure S1 for the loadings of the compositional variables). The phylogenetic clusters of the Zika strains presented in Figure 1 displayed a similar behavior for each compositional property analyzed (i.e., dinucleotide bias, RSCU, and amino acid frequencies).

Dinucleotide biases account for the dinucleotides over- and/or under-represented in a genome. For these Zika strains, the first axis (PC1) generated by the PCA accounted for 28% of the total variation, while the second axis (PC2) accounted for 18%. The results of this study are shown in Figure 2a. PC1 tended to separate the majority of clade red sequences from the rest; this axis had strong positive correlations with dinucleotides ApC and GpG, and correlated negatively with UpC and ApG. The other axis tended to distinguish blue and green clades; PC2 correlated positively with UpA and negatively with both UpG and GpU.

The redundancy of the genetic code confers the possibility to tune the efficiency and accuracy of protein production to various levels, while maintaining the same amino acid sequences [26]. The relation of codon usage among viruses and their host’s is expected to affect viral survival, fitness, evasion from the host’s immune system, and evolution [27]. PC1 accounted for 16% of the total variation, while the PC2 accounted for 14% (Figure 2b). PC1 discriminated the red clade from the green clade; this axis was explained by relatively high usage of CUC, AAU, UAC, and AUC codons, and relatively low usage of GUC, AAC, CUU, and UAU (towards the positive values of the PC1; the opposite towards the negative ones).

Amino acid frequencies vary as a result of non-synonymous mutations across a coding sequence. PC1 and PC2 accounted for 23% and 12% of the total variation, respectively (Figure 2c). PC1 separated the green clade from the other clades, due to a relative enrichment in cysteine and leucine, based on the highest correlations of this axis with the amino acid frequencies. Finally, PC2 was associated with glutamic acid and tryptophan.

### 3.3. Mapping of Amino Acid Substitutions in the ZIKV E Protein

ZIKV enters the host cell by receptor-mediated endocytosis. Importantly, the E protein has been associated with attachment and endosomal membrane fusion. Thus, the action of E-binding antibodies can impair receptor interaction and/or membrane fusion, making this protein the main target for virus-neutralizing antibodies [28]. Three domains constitute the ZIKV E protein: A central β-barrel domain (DI), an extended dimerization domain (DII), and an immunoglobulin-like segment (DIII) [29]. A fusion loop (FL) is located at the distal end of DII. This loop is inserted directly into the endosomal membrane of the host cell and then triggers fusion thanks to pH-dependent conformational changes. Furthermore, antiparallel dimers are packed in the E protein in a herringbone pattern that lies against the lipid envelope [30].

**Figure 3:**
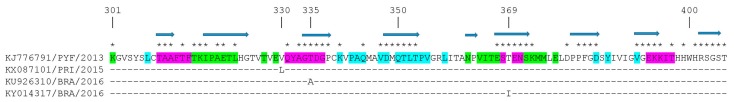
An amino acid sequence alignment of the DIII domain of ZIKV E proteins. Strains are shown by accession number, geographic location, and year of isolation. Identity of the strain H/PF/2013 from the Asian genotype (accession number KJ776791) is shown by a dash. Sequence position relative to the E protein of that strain is shown on the top of the figure. Predicted coiled regions of the protein are indicated by a blue arrow on top of the alignment. Predicted exposed residues are indicated by an asterisk on the upper part of the alignment. Previously described conformational epitopes ABDE, C-C’, and LR [30] are shown in green, blue, and magenta.

To investigate if the genetic variability observed among America´s ZIKV strains was associated with antigenic changes displayed by their E protein sequences, we explored their inferred amino acid substitutions. From the 61 strains isolated in this region, amino acid substitutions were found in only eight ZIKV strains with respect to the H/PF/2013 strain of the Asian genotype (accession number KJ776791; Table A2). Intriguingly, three substitutions were found at the DIII domain (V330L, T335A, and T369I) of E proteins from strains isolated in Latin America with respect to the H/PF/2013 strain (Figure 3).

### 3.4. Mapping Amino Acid Substitutions in the ZIKV prM Protein

**Figure 4:**
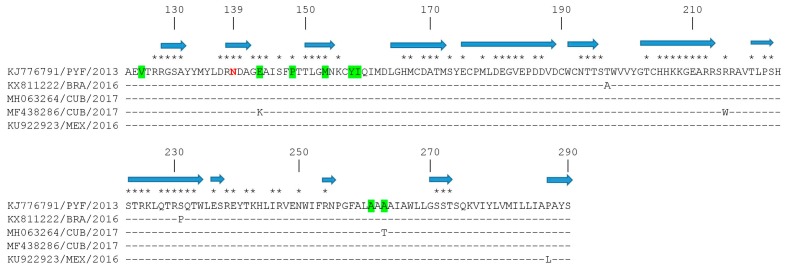
An amino acid sequence alignment of ZIKV prM proteins. Strains are shown by accession number, geographic location, and year of isolation. Identity of the strain H/PF/2013 from the Asian genotype (accession number KJ776791) is shown by a dash. Sequence position relative to the prM protein of that strain is shown on the top of the figure. Predicted coiled regions of the protein are indicated by a blue arrow on top of the alignment. Predicted exposed residues are indicated by an asterisk on the upper part of the alignment. Positions where amino acid substitutions were found between pre-epidemic and epidemic strains are highlighted in green. Position 139 is shown in red.

ZIKV virion assembly involves the interaction among prM and E proteins in the endoplasmic reticulum, the encapsulation of the RNA genome with C protein, and the coverage of a lipid bilayer containing a prM-E protein complex to form immature virions. Then, the cleavage of prM to M protein by furin or furin-like proteases in the trans-Golgi network will permit the release of mature virions [31]. Therefore, due to the important function of prM in the ZIKV life cycle, we studied the amino acid substitutions found in the ZIKV strains isolated in the American region. From the 61 strains isolated in this region, only four strains had amino acid substitutions in the prM protein, by comparison with the H/PF/2013 strain of the Asian genotype (Figure 4). All Latin American strains revealed an asparagine (N) at position 139; previous studies revealed that a serine to asparagine amino acid substitution at this position (S139N) of the ZIKV prM protein exhibited the greatest neurovirulence in neonatal mice [32]. Moreover, recent studies revealed that when pre-epidemic strains were compared with epidemic strains, several amino acid substitutions were found among pre-epidemic and epidemic ZIKV prM proteins [11,33]. Interestingly, two new substitutions were found in strains isolated in 2017 in Cuba, in positions where differences among pre-epidemic and epidemic strains were previously reported [11,33]: MF438286 had a lysine at position 143 only observed in pre-epidemic strains (substitution E143K), and MH063264 had a threonine at position 266 (substitution A266T).

## 4. Discussion

Studying the degree of genetic variability and evolution of ZIKV strains would be crucial for diagnostics, vaccine development, and disease management [9]. Previous studies have shown that ZIKV strains from the Asian genotype have evolved and spread to geographically distinct continents since approximately 1960 [34]. These studies also suggest that the strain H/PF/2013 (KJ776791) is likely the ancestor of ZIKV strains of the Asian genotype currently circulating in the American region [12,34,35]. The shape of the tree, near the root, suggested rapid early spread of the outbreak, consistent with the introduction of a new virus to an immunologically naive population. This is also in agreement with recent results [23]. ZIKV genomes from strains isolated in the Latin American region and included in these studies fell into five different genetic lineages (Figure 1). This result highlighted an important degree of diversification in the ZIKV strains in this region, in agreement with previous studies done in America [23,25].

ZIKV strains belonging to distinct genetic clades were placed at different sides in the plane formed by axes 1 and 2 of each PCA (Figure 2). The patterns shown in these figures revealed that the emergence and diversification of ZIKV in this region of the world were also reflected by their dinucleotide biases and codon usage, because the clustering in these PCA plots was comparable to the topology presented in the phylogenetic tree. Previous studies have suggested that ZIKV has evolved host- and vector-specific codon usage patterns to maintain successful replication and transmission chains within multiple hosts and vectors [36], and ZIKV strains are in the process of evolutionary fine-tuning their codon usage [37]. Moreover, selection pressure from *Homo sapiens* on the ZIKV RSCU patterns was found to be dominant compared to *Ae. aegypti* and *Ae. albopictus* vectors [38]. Recent reports identified RNA-editing by the double-stranded RNA-specific adenosine deaminase ADAR as a mechanism that may have contributed to the mutational pressure on the ZIKV genome [39], suggesting that the lower amount of areas associated with ADAR-editing in the RNA minus strand of the Asian ZIKV lineage could be the major cause behind the rise in the number of outbreaks in past decade.

Furthermore, the pattern shown by the use of amino acids (Figure 2c) reflected poorly their phylogeny. The green clade, interestingly, presented a singular behavior in their protein composition that couldn’t be explained solely by the divergence assessed in Figure 1. More studies are needed to take into account the extent of selective pressures on the ZIKV polyprotein, or in some subproducts.

Importantly, E protein is a primary antigenic target for neutralizing antibodies, which bind epitopes in all three structural domains, with many type-specific protective antibodies recognizing determinants in the DIII domain [30]. Recent studies identified the ZIKV DIII domain as a potential target for neutralizing antibodies and thus a possible immunogen for vaccines [30]. DIII has been used previously in the context of different flavivirus vaccines [40,41]. In order to discern if the diversification observed among the ZIKV strains isolated in the Latin American region may affect the antigenic structure of E proteins, we mapped the substitutions found in the E protein of ZIKV strains isolated in this region by comparison with the ZIKV strain H/PF/2013 E protein, representative of the Asian genotype [31]. Interestingly, substitutions were observed in the DIII domain of E proteins from strains isolated in this region (Figure 3). Substitution T335A mapped to a previously described DIII LR conformational epitope [30], and two of the three substitutions found mapped to exposed amino acid residues (Figure 3). More studies will be needed to address the biological relevance of these substitutions in ZIKV biology.

ZIKV has a similar structure to other known flaviviruses [42], and prM protein studies revealed that this protein is critical for viral assembly [43]. Therefore, studying the degree of genetic variability and evolution of the prM may contribute to our understanding of ZIKV infectivity and pathogenicity [13]. ZIKV prM, together with E proteins, are being used in most ZIKV vaccines currently undergoing clinical trials [44].

Among several amino acid substitutions found in ZIKV proteins, a serine to asparagine amino acid substitution (S139N) in the ZIKV prM protein, which is observed in most human epidemic strains, exhibited the greatest neurovirulence in neonatal mice [32]. These findings suggested that this serine to asparagine amino acid substitution in prM proteins of epidemic strains may have contributed to the recently observed congenital birth defects associated with ZIKV outbreaks in the American region [8]. Nevertheless, the contributions of other prM substitutions to ZIKV neurovirulence remain to be established.

When pre-epidemic and epidemic strains were compared, amino acid substitutions were identified in ZIKV prM proteins [11,33]. This genomic variability may have been driven by the adaptation of ZIKV to an urban-based transmission cycle targeting humans as hosts instead of the original sylvatic mode of transmission, as recently suggested [36]. Whether the two amino acid substitutions found in these studies at positions 21 and 140 of the prM protein may be related to these facts remains to be studied.

The relative contributions of prM amino acid substitutions to E protein structural biology are not yet well understood. However, amino acid changes may affect prM protein structure and induce structural changes in the E protein since its assembly is dependent on prM protein expression [45].

## 5. Conclusions

Altogether, these studies showed patterns of diversification of ZIKV strains circulating in the Latin American region in five different genetic groups. Moreover, different trends in dinucleotide bias and in codon usage variation among distinct genetic linages were observed, probably as a result of this diversification. Amino acid substitutions were found in E and prM proteins of ZIKV strains isolated in this region, revealing the presence of novel genetic variants circulating in the American region.

## Supplementary Materials

The following are available online at http://www.mdpi.com/1999-4915/10/12/698/s1; Table S1: Origins of the ZIKV strains; Tables S2: Compositional analyses; Figure S1: Loadings of each original variable for the first two major axes of principal component analysis.

## Appendix A

**Table A1:** Bayesian coalescent inference of ZIKV strains.

Parameter	Value ^a^	HPD ^b^	ESS ^c^
Log likelihood	−21,802.85	−21,852.01 to −21,744.02	3179.90
Clock rate ^d^	1.21 × 10^−3^	7.55 × 10^−4^ to 1.66 × 10^−3^	4823.21
tMRCA ^e^ All	4.20	3.70 to 5.03	291.75
	**29/6/2013**	**3/10/2012 to 2/13/2014**	
tMRCA ^e^ Latin American clade	3.37	2.87 to 6.33	
	**3/27/2014**	**10/24/2013 to 11/13/2014**	

^a^ In all cases, the mean values are shown; ^b^ HPD, highest posterior density values; ^c^ ESS, effective sample size; ^d^ clock rate was calculated in substitutions/site/year; ^e^ tMRCA, time of the most common recent ancestor is shown in years. The date estimated is indicated in bold.

## Appendix B

**Table A2:** Mapping of amino acid substitutions in E proteins of ZIKV strains isolated in Latin America.

Strain ^a^	Amino Acid Position ^b^							
	23	51	68	260	330	335	369	443
KU321639/BRA/2015	I							
MH157202/MEX/2016		T						
MF801378/GMT/2016			T					
KU497555/BRA/2015				T				
KX087101/PRI/2015					L			
KU926310/BRA/2016						A		
KY014317/BRA/2016							S	
KX694534/HND/2015								R

^a^ Strains are shown by accession number, geographic location, and date of isolation; ^b^ amino acid positions are relative to strain H/PF/2013 (KJ776791) of the Asian genotype.
