# Supplementary material for: An Evolutionary Insight into Zika Virus Strains Isolated in the Latin American Region
Source: Viruses. 2018 Dec 8;10(12):698. doi: 10.3390/v10120698 (PMC6316622; doi:10.3390/v10120698)
Supplement: Supplementary file 1 [file viruses-10-00698-s001.zip › viruses-373334-rev2-Supplementary_Material_Table_S1.docx]

**Supplementary Material Table S1.** Origins of the ZIKV strains.

Accession number Geographic location Date of isolation

KU527068 Brazil (BRA) 2015

KU321639 “ 2015

KU497555 “ 2015

MF073357 “ 2016

KY441402 “ 2016

KY441403 “ 2016

KU926309 “ 2016

KU926310 “ 2016

KX811222 “ 2016

KY014301 “ 2016

KY014317 “ 2016

KY272991 “ 2016

KX087102 Colombia (COL) 2015

KX247646 “ 2016

MH063262 Cuba (CUB) 2017

MH063264 “ 2017

MF438286 “ 2017

MF664436 Dominican Republic (DOM) 2016

KU853012 “ 2016

KU853013 “ 2016

KY014321 “ 2016

KY785415 “ 2016

KY785420 “ 2016

MF794971 Ecuador (ECU) 2016

KX879603 “ 2016

KX879604 “ 2016

KJ776791 French Polynesia (PYF) 2013

MF801378 Guatemala (GTM) 2016

MF593625 “ 2016

KU509998 Haiti (HTI) 2014

KX051563 “ 2016

KX269878 “ 2016

KX694534 Honduras (HND) 2015

MF801387 “ 2016

KX906952 “ 2016

KY785418 “ 2016

KY014315 “ 2016

**Supplementary Material Table S1 (Cont.).** Origins of the ZIKV strains.

Accession number Geographic location Date of isolation

MH157202 Mexico (MEX) 2016

MH157208 “ 2016

MH157213 “ 2016

KU922923 “ 2016

KU922960 “ 2016

KY120348 “ 2016

KY120349 “ 2016

MF801426 Nicaragua (NIC) 2016

MF434517 “ 2016

KY765317 “ 2016

KY765318 “ 2016

KY765320 “ 2016

KY765323 “ 2016

KY765324 “ 2016

KY765325 “ 2016

KX156774 Panama (PAN) 2015

KX156775 “ 2015

KX156776 “ 2015

KY693679 Peru (PER) 2016

KY693678 “ 2016

KX601168 Puerto Rico (PRI) 2015

KX087101 “ 2015

KY785464 “ 2016

KY325464 USA 2016

KY325465 “ 2016

KY325468 “ 2016

KY325469 “ 2016

KY325472 “ 2016

KY325473 “ 2016

KY325476 “ 2016

KY325477 “ 2016

KY325479 “ 2016

KY693680 Venezuela (VEN) 2016

KX702400 “ 2016

KX893855 “ 2016
