# Supplementary material for: An Evolutionary Insight into Zika Virus Strains Isolated in the Latin American Region
Source: Viruses. 2018 Dec 8;10(12):698. doi: 10.3390/v10120698 (PMC6316622; doi:10.3390/v10120698)
Supplement: Supplementary file 1 [file viruses-373334_rev2-Supplementary_Material_Figure_S1.pdf › viruses-373334_rev2-Supplementary_Material_Figure_S1.pdf]

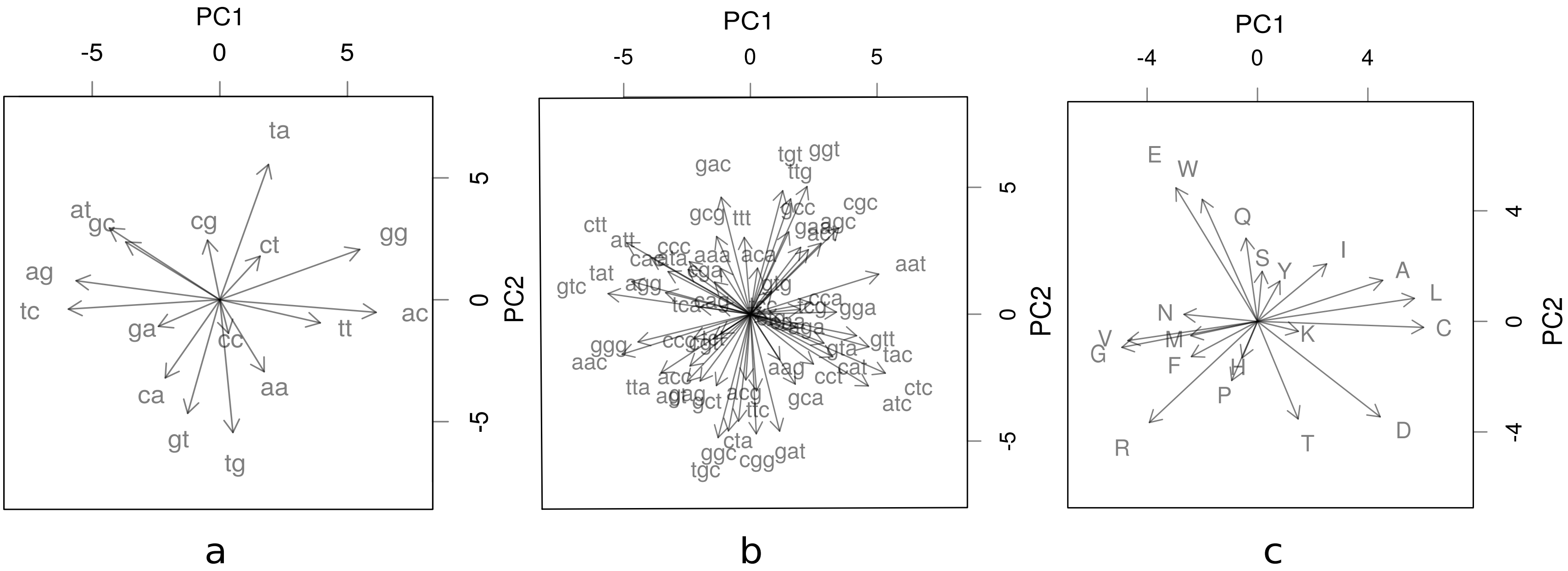

Figure S1. Loadings of each original variable for the first two major axes of principal component analysis of (a) dinucleotide observed/expected ratios, (b) relative synonymous codon usage, and (c) amino acid frequencies.
